# Cross-Subject Motor Imagery Electroencephalogram Decoding with Domain Generalization

**DOI:** 10.3390/bioengineering12050495

**Published:** 2025-05-07

**Authors:** Yanyan Zheng, Senxiang Wu, Jie Chen, Qiong Yao, Siyu Zheng

**Affiliations:** 1 Department of Neurology, Wenzhou Third Clinical Institute Affiliated to Wenzhou Medical University, Wenzhou People’s Hospital, Wenzhou 325000, China; wusenxiang8@163.com (S.W.); 13736744198@163.com (Q.Y.); 2 Department of Pediatrics, Wenzhou Third Clinical Institute Affiliated to Wenzhou Medical University, Wenzhou People’s Hospital, Wenzhou 325000, China; 3 Shanghai Shaonao Sensing Technology Co., Ltd., Shanghai 200444, China; 19802129927@163.com

**Keywords:** brain–computer interface, deep learning, transfer learning

## Abstract

Decoding motor imagery (MI) electroencephalogram (EEG) signals in the brain–computer interface (BCI) can assist patients in accelerating motor function recovery. To realize the implementation of plug-and-play functionality for MI-BCI applications, cross-subject models are employed to alleviate time-consuming calibration and avoid additional model training for target subjects by utilizing EEG data from source subjects. However, the diversity in data distribution among subjects limits the model’s robustness. In this study, we investigate a cross-subject MI-EEG decoding model with domain generalization based on a deep learning neural network that extracts domain-invariant features from source subjects. Firstly, a knowledge distillation framework is adopted to obtain the internally invariant representations based on spectral features fusion. Then, the correlation alignment approach aligns mutually invariant representations between each pair of sub-source domains. In addition, we use distance regularization on two kinds of invariant features to enhance generalizable information. To assess the effectiveness of our approach, experiments are conducted on the BCI Competition IV 2a and the Korean University dataset. The results demonstrate that the proposed model achieves 8.93% and 4.4% accuracy improvements on two datasets, respectively, compared with current state-of-the-art models, confirming that the proposed approach can effectively extract invariant features from source subjects and generalize to the unseen target distribution, hence paving the way for effective implementation of the plug-and-play functionality in MI-BCI applications.

## 1. Introduction

An electroencephalogram (EEG) is a medical imaging technique that detects scalp electrical activity generated by brain structures through metal electrodes [1]. The noninvasive nature and high temporal resolution of EEGs [2] have made EEG-based brain–computer interfaces (BCIs) widely applicable in multiple fields such as disease diagnosis [3,4,5], robot control [6,7,8], and other fields [9], especially in rehabilitation applications [10,11,12]. Motor imagery (MI) is recognized as one of the most significant BCI exponents, enabling individuals with disabilities to control EEG signals without external stimulation. By decoding EEG signals from the associated motor cortex on the brain scalp, motor intentions can be recognized, thus facilitating patients’ proactive engagement in the rehabilitation stage [13].

Traditional machine learning methods such as common spatial pattern (CSP) [14] have made significant progress in MI task classification by constructing optimal filters to extract spatial features. Variants of the CSP, like common spatio-spectral pattern (CSSP) [15] and sub-band common spatial pattern (SBCSP) [16], further improve classification accuracy by improving time domain feature extraction and the frequency band selection. The filter bank common spatial pattern (FBCSP) [17] has been used to extract the optimal features of several band-pass filters with various band ranges and has been well verified on different datasets. The common classifiers used in the MI-BCI field are linear discriminant analysis (LDA) and support vector machines (SVM) [18]. Despite the remarkable achievements that have been achieved, the insufficient merging of feature extraction methods and classifiers presents an issue with the model’s accuracy, resilience, and flexibility.

Deep learning (DL), an end-to-end approach for decoding and encoding signals, has been applied successfully in MI-EEG task classification. Classical models, such as shallow ConvNet and deep ConvNet designed by Schirrmeister et al. [19], have reached accuracies in the same range compared with FBCSP for the first time. The structure for capturing temporal–spatial features from EEGs based on the convolutional neural network (CNN) and model design choices such as batch normalization and exponential linear units (ELU) have proven to be crucial for classification. Based on the structure of shallow ConvNet, EEGNet proposed by Lawhern et al. [20] uses a separable depthwise CNN layer to reduce the dimensionality. The model successfully extended EEG application scenarios while ensuring high accuracy. To further enhance performance and reduce parameters, Mane et al. [21] introduced a multi-view deep learning model called the filter-bank convolutional network (FBCNet). This model was designed to capture optimized spectral representations from MI-EEG using a range of spectral filtering techniques, similar to the procedures in FBCSP. The attention mechanism, widely employed in the deep learning field, also plays a significant role in EEG decoding. Li et al. [22] extracted attention-based features by adopting the squeeze-and-excitation network (SENet). Song et al. [23] constructed a hybrid model employing six transformer encoders [24] based on multi-head attention after CNN layers, and it performed well in the hold-out scenario. DL-based models have shown excellent performance on MI-EEG decoding. However, DL applications are usually limited by the long training time, high resource consumption, and a heavy reliance on the number of labeled data [25]. In practical BCI applications, it is a challenge to collect sufficient data with good quality to build individualized models for each person. Meanwhile, achieving the goal of immediate usability with DL approaches is hard for patients because models require a significant amount of time for training to achieve a high classification accuracy. Therefore, there is a strong desire to recognize patients’ MI intentions without additional experimental data collection and modeling.

Domain adaptation (DA) approaches are the specialized instances of transfer learning (TL) where a model trained on the source domain is adapted or fine-tuned to perform well on a different but related domain, namely the target domain [26]. DA-based approaches using source data for the pretraining model and part of data from the target domain for optimizing are becoming widely applied in real MI-EEG applications. For instance, Chen et al. [27] combined a support matrix machine (SMM) with knowledge leverage (KL) to learn transferable knowledge by integrating the model knowledge from the source domain and a part of the target domain. Wei et al. [28] performed data distribution alignment for each subject in the source domain with the target one and integrated the outcomes through decision fusion. Liang et al. [29] employed a balanced distribution adaptation algorithm to minimize the distribution distance by selecting source subjects based on the similarity of spatial covariance matrices in the Riemannian space. Compared with instance-based methods, feature-based methods preserve the properties or potential structures of the data and facilitate the identification of correlations between features by constructing a new feature representation [30]. Hang et al. [31] used maximum mean discrepancy (MMD) to minimize the distribution discrepancy and force thd deep features closer to the corresponding class centers using center-based discriminative feature learning. Chen et al. [32] adopted the gradient reversal layer (GRL) based on the adversarial structure to extract common features among the source and target domains. Hong et al. [33] designed a DL network with two domain label classifiers to dynamically evaluate the joint of marginal and conditional discrepancy. The other parameter-based methods based on fine-tuning technology also achieve impressive progress [34,35]. Nevertheless, instance-based adaptation approaches frequently rely on conventional machine learning techniques for binary classification tasks, imposing the challenge of time-consuming selection of appropriate paired data from the source domain. Similarly, other domain adaptation methods demand the collection of data from the target domain and the construction of models, which fail to align with the expectations of patients for a ‘ready-to-use’ solution.

Compared with DA, domain generalization (DG) approaches only consider the data from the source domains and develop models that can generalize to unfamiliar distributions. Given the limited real training data, a simple way to enhance the generalization capability is to create more manual data. For instance, Tobin et al. [36] added domain randomization for generalization in the real environment by changing the number, shape, texture, and other characteristics of the objects. Zhang et al. [37] proposed a data generation-based DG method, namely Mixup, to generate new training samples by linearly blending the features and labels of different data. Another group of methods is representation learning, which adopts kernels, adversarial training, or feature alignments to learn domain invariant representations [38]. Grubinger et al. [39] employed transfer component analysis (TCA) [40] to understand a common subspace by reducing the disparities among domains. The approaches like domain-invariant component analysis (DICA) [41] and scatter component analysis (SCA) [42] are also classical kernel-based methods similar to the idea of TCA. Li et al. [43] extracted domain-invariant features through adversarial losses that consider the source–domain label information. In the BCI field, conventional data augmentation-based DG techniques, including sliding windows [19], adding noise, over-sampling [44], and geometric transformation [45], have shown improvement in classification accuracy. While these methodologies constitute valid domain generalization strategies, comparatively less attention has been directed toward feature-centric optimization strategies. Moreover, the inter- and intra-subject variability constrains the models’ generalization capacity, so previous studies primarily focused on constructing within-subject models and not fully harnessing cross-subject data within the source domain [46]. Therefore, DG-based models remain largely unexplored and have not yet reached the capability to provide a calibration-free BCI solution for real-world applications. Wang et al. [47] utilized knowledge distillation to extract invariant features from pictures in the computer vision field. Inspired by its framework, we apply knowledge distillation in our work to extract the cross-domain representations in MI-EEG signals.

In this paper, we propose a cross-subject model with a DG approach. The dataset is divided into a source domain consisting of several subdomains and a target domain. The data in the target domain with the unseen distributions will not be involved in the model’s training and validation. The proposed model improves the domain generalization ability by extracting the internal and mutually invariant features among different subjects. A knowledge distillation framework is employed to capture the spectral information of EEG signals as internally invariant representations. For mutually invariant features, the correlation alignment (CORAL) [48] method is used to align the feature distributions between any two subdomains from the source data. To reduce the possible redundancy between the internal and mutual features, the proposed model utilizes a regularization technique to enhance their dissimilarity. In the model training phase, the early stopping (ES) technology and the two-stage training strategy are used to prevent model overfitting and fully utilize all source domain data. We conduct comprehensive experiments on two MI-EEG datasets to prove the excellent generalization capability of the proposed model.

The remainder of the paper is outlined as follows. The data description, preprocessing steps and detailed model structure are presented in Section 2. The experiments and results are detailed in Section 3. Then, the discussion is presented in Section 4. Finally, Section 5 concludes the paper.

## 2. Methods

### 2.1. Definitions

In domain generalization, *X* denotes an input space for EEG signals, and *Y* is an output space. The domain is defined as $S={\left\{\left(x_{i},y_{i}\right)\right\}}_{i=1}^{n}∼P_{XY}$, where $P_{XY}$ denotes the joint distribution and x∈X,y∈Y. The source domain with labeled data is divided into multiple training subdomains, namely $S_{train}=\left\{S^{i}\left|i=1,\ldots ,N\right.\right\}$, where *N* is the number of subdomains, and $S^{i}=\left\{{\left(x_{j}^{i},y_{j}^{i}\right)}_{j=1}^{n_{i}}\right\}$ represents the $i^{th}$ subdomain. In the real scenario of MI-EEG classification, the internal and external diversities among subjects make the joint distributions between each pair of sub-source domains different: $P_{XY}^{i}\neq P_{XY}^{j},1⩽i\neq j⩽M$. According to [38], domain generalization aims to acquire a resilient and broadly applicable predictive function f:X⟶Y from the N subdomains to minimize errors when applied to an unseen test domain $S_{test}\left(i.e.,P_{XY}^{test}\neq P_{XY}^{i}\;\;for\;\;i\in \left\{1,\ldots ,N\right\}\right)$:

(1) $$\min_{f}E_{\left(x,y\right)\in S_{test}}\left[loss\left(f\left(x\right),y\right)\right],$$

where *E* is the expectation, and loss is the loss function. Differing from domain adaptation methods, data from $S_{test}$ will not be involved in the training and validation processes.

### 2.2. Framework

**Figure 1:**
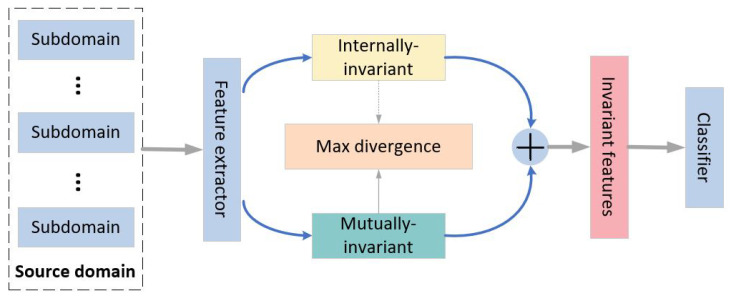
The framework of the proposed model.

The EEG dataset consists of the source domain and the target domain. The source domain is divided into multiple subdomains sent into the proposed model, as shown in Figure 1. Then, internally and mutually invariant representations are captured through a feature extractor. To differentiate these two kinds of information, a regularization technique is adopted by maximizing the divergence. In the end, the invariant features are concatenated together for classification.

### 2.3. Internally Invariant Features

**Figure 2:**
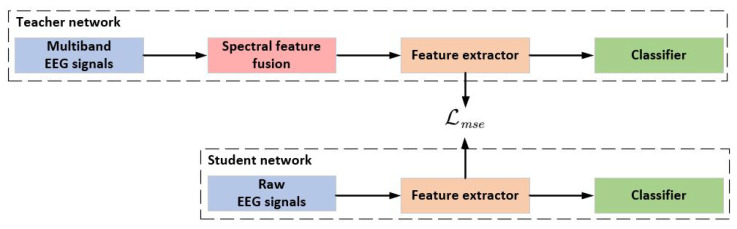
The framework of distillation to learn internally invariant features.

**Figure 3:**
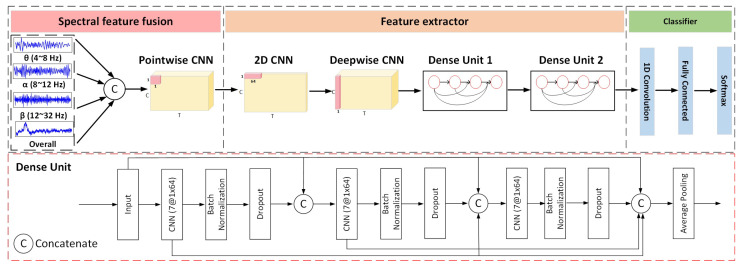
The model structure of the teacher network.

Previous studies [49,50] have revealed that the most frequently utilized frequency bands in MI-EEG research are the α rhythm, typically around 10 Hz, and the β rhythm, typically around 20 Hz. In [51,52], the θ rhythm with a range of 4 to 7 Hz was incorporated and demonstrated its utility in decoding MI-EEG signals. Although the appropriate operational frequency bands vary from person to person [16], the information utilized for conducting the imagination classification task is primarily concentrated within these sub-bands. Hence, the spectral features based on multi-band EEG signals are employed as internally invariant representations in the source domain. Knowledge distillation is a straightforward framework for promoting specific characteristics within different networks [47]. The distillation framework consists of the teacher and the student network (Figure 2). The teacher network fuses the spectral information for MI classification and guides the student network to learn invariant information. The structure of the teacher network, composed of three components, is shown in Figure 3.

#### 2.3.1. Spectral Feature Fusion

We select three sub-bands, θ (4–7 Hz), α (7–13 Hz), and β (13–32 Hz), and the overall band as the inputs sent into the teacher model across our previous work [53]. The study [54] proves the robustness of spectral representation for MI tasks can be enhanced by adopting cross-frequency interactions. Therefore, we concatenated the filtered data in the feature dimension to associate multiple-frequency neural oscillations. The $i_{th}$ single-trial EEG sample is defined as $X_{i}\in R^{C\times T}$, where C represents channels and T represents time points. The fused multi-band EEG data $X_{MB}$ is determined as follows:

(2) $$X_{MB}=X\times h\left(n\right)\in R^{N_{b}\times C\times T},$$

where $h\left(n\right)$ denotes the three-order Butterworth filter corresponding to the $n_{th}$ frequency sub-band, and $N_{b}$ is the number of sub-bands. The pointwise CNN, subsequently utilized, performs convolution on each time point and channel of the EEG data. The output dimension is set to one so that the complementary information available in each frequency band is fused. Additionally, it assigns an adaptive weight to each frequency band, reducing noise in redundant frequency bands while enhancing valuable information in other frequency bands.

#### 2.3.2. Feature Extractor

Following the fusion of spectral features, we utilize two convolution layers to learn discriminative temporal–spatial information [19,20,21]. The first CNN layer using a $1\times k_{t}$ kernel is employed for the EEG channel to extract temporal features. The value of $k_{t}$ is equal to one-fourth of the data sampling rate, enabling the capture of frequency information at 4 Hz and beyond [20]. Then, we use a $k_{s}\times 1$ depthwise CNN to extract spatial features across all selected EEG channels. The kernel size $k_{s}$ is configured to match the number of channels, allowing the compression of data collected at each time step into a single feature map. This strategy leads to a decrease in model parameters and enhances efficiency.

To further extract useful information from the temporal–spatial features, two dense units consisting of several CNN and pooling layers are applied subsequently (Figure 3). Suppose that the network comprises a total of *L* layers, with each layer utilizing a non-linear function $F_{l}\left(\cdot \right)$, where *l* represents the layer index, and the output of each layer is denoted as $x_{l}$. A common transformation involving a single path between each operation layer is the following:

(3) $$x_{l}=F_{l}\left(x_{l-1}\right).$$

As the network becomes deeper and wider, parts of useful features are filtered. Additionally, an abundance of training parameters can result in significant overfitting issues, particularly when dealing with MI-EEG signals that contain a considerable amount of noise and redundant information. To tackle this issue, we establish short connections from any given layer to all subsequent ones. For instance, the *l*th layer obtains the feature maps from all preceding layers:

(4) $$x_{l}=F_{l}\left(\left[x_{0},x_{1},\ldots ,x_{l-1}\right]\right),$$

where $\left[x_{0},x_{1},\ldots ,x_{l-1}\right]$ are the feature maps from the preceding CNN layers [l0,l1,…,l−1]. In the case where a CNN layer updates *k* feature matrices, the *l*-th layer encompasses a total of $k_{0}+k\times \left(l-1\right)$ inputs. $k_{0}$ represents the raw dimension in the input layer, while *k* signifies the growth rate, indicating the extent to which further knowledge is acquired and transmitted to the subsequent layer. As a result, the connections from the preceding layers substantially increase to $\frac{L(L+1)}{2}$, in contrast to the traditional transition, where only *L* connections are made in a network comprising *L* layers. Every layer obtains access to all the feature maps from preceding layers, facilitating improved information propagation and utilization of features. The ELU function is adopted as activation to reduce gradient explosion and increase model robustness. The following Batch normalization and dropout techniques help to reduce overfitting risks.

#### 2.3.3. Classifier

The classifier includes a 1D CNN, a fully connected layer, and a dense layer with the softmax function for classifying MI tasks. The fused multi-band EEG signals $\tilde{x}\in X_{MB}$ and the corresponding label *y* are sent to the teacher network for training:

(5) $$\underset{\theta_{T}^{f},\theta_{T}^{c}}{\begin{array}{c} \min\; \end{array}}E_{\left(\tilde{x},y\right)∼P^{tr}}L_{cls}\left(G_{T}^{c}\left(G_{T}^{f}\left(\tilde{x}\right)\right),y\right),$$

where $\theta_{T}^{f}$ and $\theta_{T}^{c}$ are the parameters of feature extractor $G_{T}^{c}$ and the classifier $G_{T}^{f}$ in the teacher network. *E* is the expectation, while $P^{tr}$ represents the data distribution in the source domain. The loss function $L_{cls}$ is the cross-entropy loss, which quantifies the difference between the probability distribution of the model predictions represented as $y_{p}$ and the real labels denoted as $y_{t}$:

(6) $$L_{cls}\left(y_{p},y_{t}\right)=-\sum_{m}y_{p,m}\log y_{t,m},$$

where *m* is the index of *y*. After training and optimizing the teacher network, we use the obtained features from the teacher network to guide the student network to learn the spectral invariant representations:

(7) $$\begin{array}{c} \underset{\theta_{S}^{f},\theta_{S}^{c}}{\begin{array}{c} \min\; \end{array}}E_{\left(\tilde{x},y\right)∼P^{tr}}L_{cls}\left(G_{S}^{c}\left(G_{S}^{f}\left(x\right)\right),y\right)\\ +\lambda_{1}L_{mse}\left(G_{S}^{f}\left(x\right),G_{T}^{f}\left(\tilde{x}\right)\right), \end{array}$$

where $\theta_{S}^{f}$ and $\theta_{S}^{c}$ are the parameters of feature extractor $G_{S}^{c}$ and the classifier $G_{S}^{f}$ in the teacher network. $lambda_{1}$ is an adjustable hyperparameter to limit the mean squared error (MSE) $L_{mse}$, which brings the features of the student network into proximity with those of the teacher network:

(8) $$L_{mse}=\frac{1}{n}\sum_{i=1}^{n}{\left({\hat{y}}_{i}-y_{i}\right)}^{2},$$

where *n* is the index of *y*. Full details of the network structure are presented in Table 1. The parameters used in dense unit 1 are the same as unit 2; hence, specific details are not displayed in the table. The difference between the student and teacher networks lies in the absence of spectral feature fusion in the student network. Additionally, in the classifier block, parameter F3 is twice the size of the one in the teacher network in order to encompass two types of invariant features simultaneously.

### 2.4. Mutually Invariant Features

The student network learns the invariant spectral features from the teacher network by the knowledge distillation framework to classify MI tasks. However, it disregards the discrepancies in data distribution among subdomains, which means that internally invariant features alone are insufficient to guarantee excellent generalization capability. To learn the invariant representations from the source domain, the correlation alignment approach is employed to align the second-order statistics of the features from any two domains:

(9) $$L_{align}=\frac{2}{N\times \left(N-1\right)}\sum_{i\neq j}^{N}{∥C_{i}-C_{j}∥}_{F}^{2},$$

(10) $$C_{i}=\frac{1}{n_{i}-1}\left({X_{i}}^{T}X_{i}-\frac{1}{n_{i}}{\left(1^{T}X_{i}\right)}^{T}\left(1^{T}X_{i}\right)\right),$$

where $C_{i}$ represents the covariance matrix. Internally invariant features primarily highlight spectral information for MI task classification, while mutually invariant features center around cross-domain representations. To better represent these two kinds of features, the outputs of the 1D CNN layer in the student network are divided into internally invariant features $z_{1}$ and mutually invariant features $z_{2}$. Before feeding to the final classification layer, we expect to reduce redundant information and make it more diversified between $z_{1}$ and $z_{2}$. Thus, we use the regularization tool to maximize their divergence:

(11) $$L_{div}\left(z_{1},z_{2}\right)=-d\left(z_{1},z_{2}\right),$$

where $d\left(.\right)$ denotes the L2 distance: $L_{div}=-∥{z_{1}-z_{2}∥}_{2}^{2}$. In summary, the aim of the student network is established as follows:

(12) $$\begin{array}{cc} \underset{\theta_{S}^{f},\theta_{S}^{c}}{\begin{array}{c} \min \end{array}} & E_{\left(\tilde{x},y\right)∼P^{tr}}L_{cls}\left(G_{S}^{c}\left(G_{S}^{f}\left(x\right)\right),y\right)\\ \; & +\lambda_{1}L_{mse}\left(z_{1},G_{T}^{f}\left(\tilde{x}\right)\right)\\ \; & +\lambda_{2}L_{align}+\lambda_{3}L_{div}\left(z_{1},z_{2}\right) \end{array},$$

where $\lambda_{1}$, $\lambda_{2}$, and $\lambda_{3}$ are hyperparameters to limit the contribution of each loss function.

## 3. Experiments and Results

### 3.1. Datasets

#### 3.1.1. Dataset I

The BCI Competition IV 2a (BCIC-IV-2a) dataset, as described in [55], consists of nine healthy subjects with four distinct MI tasks: left-hand, right-hand, both-feet, and tongue movements. The EEG data were captured using 22 EEG electrodes at a sampling rate of 250 Hz. Then, signals underwent bandpass filtering within the range of 0.5 Hz to 100 Hz, along with notch filtering at 50 Hz. Each subject participated in two separate recording sessions on different days, and each session consisted of 288 trials. All sessions were categorized within either the source domain or the target domain.

#### 3.1.2. Dataset II

The Korean University (KU) dataset [56] is one of the largest MI datasets, comprising EEG signals from fifty-four healthy subjects. Every subject engaged in 200 trials, with 100 trials dedicated to the left-hand MI task and another 100 to the right-hand MI task. EEG signals were captured from 62 EEG electrodes and initially sampled at a rate of 1000 Hz. To facilitate equitable comparisons with other techniques, we resampled the raw signals to 250 Hz. Subsequently, 20 channels were chosen from the region associated with motor function based on the previous study [21].

### 3.2. Training Procedure

**Figure 4:**
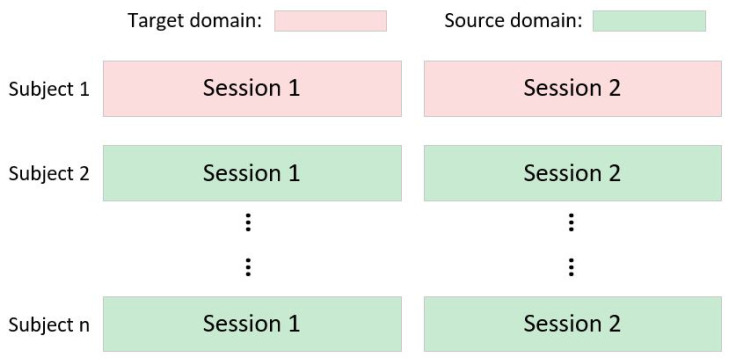
The experimental settings of the “leaving one subject out” strategy.

The “leaving one subject out” (LOSO) strategy (Figure 4) was used in our experiment. One subject was selected as the test set in the target domain. The remaining subjects were sent into the source domain. The subjects in the source domain were divided into *k* groups, with each group serving as a sub-source domain. To take full advantage of all the data in the source domain, we employed a two-stage training strategy according to [21] and the early-stopping (ES) technique. First, all data were divided into two parts, with 80% designated for training and 20% for validation. Five-fold cross-validation was employed in the first training stage. The ES technique regards the validation loss as the criterion and monitors every epoch. Training was terminated when the loss of the validation set did not decrease within a specified number of ES epochs or the number of training epochs exceeded the predefined threshold value. Once the model with the highest validation accuracy was built, the corresponding validation loss was also recorded. Then, to involve all the source domain data in the training process, the model built in the first stage was trained again using both the training and validation sets. The validation loss was monitored by the ES. If it fell below the previously recorded loss in stage one, training was stopped. In order to ensure the model’s convergence, a maximum limit of 1000 training epochs was imposed for stage one and 400 for stage two. The Adam optimizer was adopted. In the first stage, the learning rate was configured as 0.001. In the second stage, if the number of epochs was less than 150, the learning rate remained at 0.001. However, if the number of epochs exceeded 150, the learning rate was adapted to $1\times 10^{-4}$.

The computer system utilized in this experiment was equipped with 22AMD processors (manufactured by TSMC) and 90 GB of RAM. For training and testing EEG data, a GTX 4090 GPU with 24 GB of memory was employed. The proposed model and baseline models were constructed using PyTorch 1.9.0 based on Python 3.8.

### 3.3. Baseline Models

The proposed model was compared with the following benchmarks: traditional machine learning approaches (CSP [14] and FBCSP [17]), CNN-based approaches (shallow ConvNet [20], EEGNet [20], and FBCNet [21]), and their variants [57] based on dynamic CNNs.

#### 3.3.1. Machine Learning Approaches

CSP and FBCSP are the most commonly used benchmark models in the traditional machine learning domain. CSP determines the optimal spatial filters by diagonalizing a matrix for data mapping. Building upon the effective extraction of spatial features, FBCSP can mitigate the influence of subject-specific variations in frequency bands by identifying discriminative pairs of them. As described in [17], EEG signals are decomposed into nine frequency bands, each spanning a 4 Hz range from 4 to 40 Hz, utilizing Chebyshev filters in the FBCSP model. For classification, the support vector machine (SVM) with the default radial bias function (RBF) kernel is employed.

#### 3.3.2. CNN-Based Approaches

Shallow ConvNet first used CNN layers to extract temporal–spatial features from the EEG signals. The log, square, and pooling operations are adopted to deal with features. Based on this shallow structure, EEGNet utilizes a separable CNN layer to refine temporal–spatial features, making it suitable for classification tasks across various EEG data while ensuring the quality of classification. FBCNet refers to the core idea of the FBCSP, dividing the EEG signals into nine sub-frequency bands ranging from 4 to 40 Hz. Each sub-band is fed into the model to capture spatial features. A variance layer is employed with a fully connected layer following to unite features. All three models exhibit excellent performance and robustness for within-subject and cross-subject scenarios of primary MI-EEG datasets.

#### 3.3.3. Dynamic CNN-Based Approaches

Barmpas et al. [57] present a framework built upon dynamic convolutions, incorporating the subject attention network. This framework, with no need for calibration, effectively addresses the challenge of variability caused by the data distribution drift. Shallow ConvNet, EEGNet, and the EEG-inception network, aided by dynamic CNNs, have demonstrated favorable classification performance and robustness in cross-subject scenarios on the KU dataset. We also utilize their results as a point of comparison.

### 3.4. Experimental Results

The averaged classification accuracy of different methods is shown in Table 2 and Table 3. Statistical significance tests between benchmarks and the proposed method were conducted. For BCIC-IV-2a dataset, the results obtained by various methods are as follows: 35.09% (p<0.01) for CSP, 35.45% (p<0.01) for FBCSP, 51.14% (p<0.05) for shallow ConvNet, 43.42% (p<0.01) for EEGNet, 41.27% (p<0.01) for FBCNet, and 60.07% for our proposed model. The proposed method surpasses the best benchmark result by 8.93%. In the KU dataset, the results achieved by different methods are as follows: 56.08% (p<0.01) for CSP, 65.19% (p<0.01) for FBCSP, 74.62% (p<0.01) for shallow ConvNet, 72.23% (p<0.01) for EEGNet, 71.54% (p<0.01) for FBCNet, 70.30% (p<0.01) for dynamic shallow ConvNet, 71.90% (p<0.01) for dynamic EEGNet, 77.40% (p<0.01) for dynamic EEG-inception, and 81.80% (p<0.01) for our proposed model. The proposed method outperforms the best benchmark result by 4.4%. The results tested on two datasets demonstrate that our proposed model effectively decodes EEG signals and extracts useful cross-domain information from source data. The trained model successfully achieved excellent classification results in the unseen target domain.

### 3.5. Ablation Study

The proposed model used the knowledge distillation framework and feature alignment method to capture internally and mutually variant representations. A regularization technique was adopted to separate two kinds of features. To validate the contributions of each component, an ablation experiment was conducted by controlling the losses $L_{mse}$, $L_{align}$, and $L_{div}$ in Equation (12). The classification results based on the proposed model without internally invariant features (w./o Inter), without mutually invariant features (w./o Mutual), without the divergence maximum between two invariant features (w./o Div), and without the whole generalization improvement part (w./o General) are shown in Table 4. Any missing component will indeed lead to a decrease in the accuracy of the proposed model. Among them, the performance of w./o Div drops more significantly than other cases in both datasets, indicating the necessity to maximize the divergence of two invariant features.

### 3.6. Parameter Sensitivity

**Figure 5:**
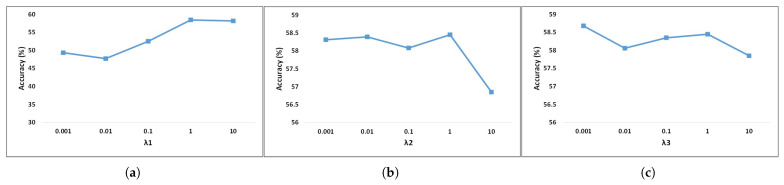
Parameter sensitivity in the loss function (BCIC-IV-2a dataset). (**a**) depicts $\lambda_{1}$, (**b**) depicts $\lambda_{2}$, (**c**) depicts $\lambda_{3}$.

**Figure 6:**
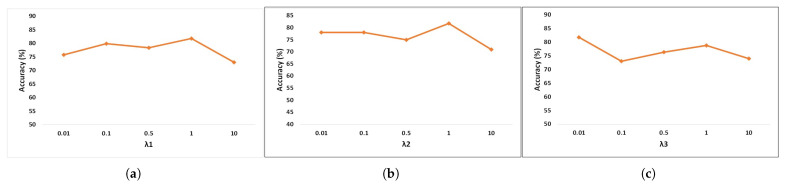
Parameter sensitivity in the loss function (KU dataset). (**a**) depicts $\lambda_{1}$, (**b**) depicts $\lambda_{2}$, (**c**) depicts $\lambda_{3}$.

**Figure 7:**
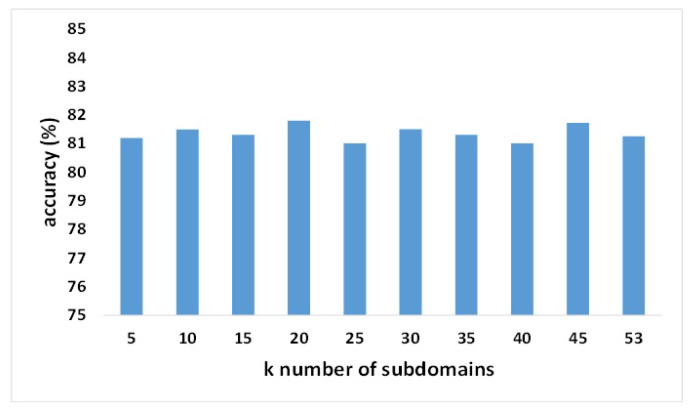
Parameter sensitivity of the number of subdomains (KU dataset).

The hyperparameters $\lambda_{1}$, $\lambda_{2}$, and $\lambda_{3}$ are adjustable in the experiment. In Equation (12), $\lambda_{1}$ limits the contribution of $L_{mse}$ to the loss function, $\lambda_{2}$ for $L_{align}$, and $\lambda_{3}$ for $L_{div}$. In the experiment, we fixed two of them and changed the rest in the two datasets. The results are exhibited in Figure 5 and Figure 6. When the value of a hyperparameter exceeds one, it significantly amplifies the contribution of a specific loss to the overall loss, resulting in an excessively large final loss value, which negatively impacts the model’s accuracy. Furthermore, the number of subdomains is also an alterable hyperparameter. We randomly divided all subjects from the source domain into several groups, ensuring that the number of subjects within each group was similar. Each group served as a separate subdomain. The BCIC-IV-2a dataset only had nine subjects; hence, we divided the source domain, including eight subjects, into eight subdomains. The source domain of the KU dataset had 53 subjects, so we split them into k groups and tested the influence of the number of subdomains. As shown in Figure 7, the averaged accuracies of the proposed model remained stable with different numbers of subdomains. We chose k=20 in the KU dataset to obtain the best performance.

### 3.7. Visualization

**Figure 8:**
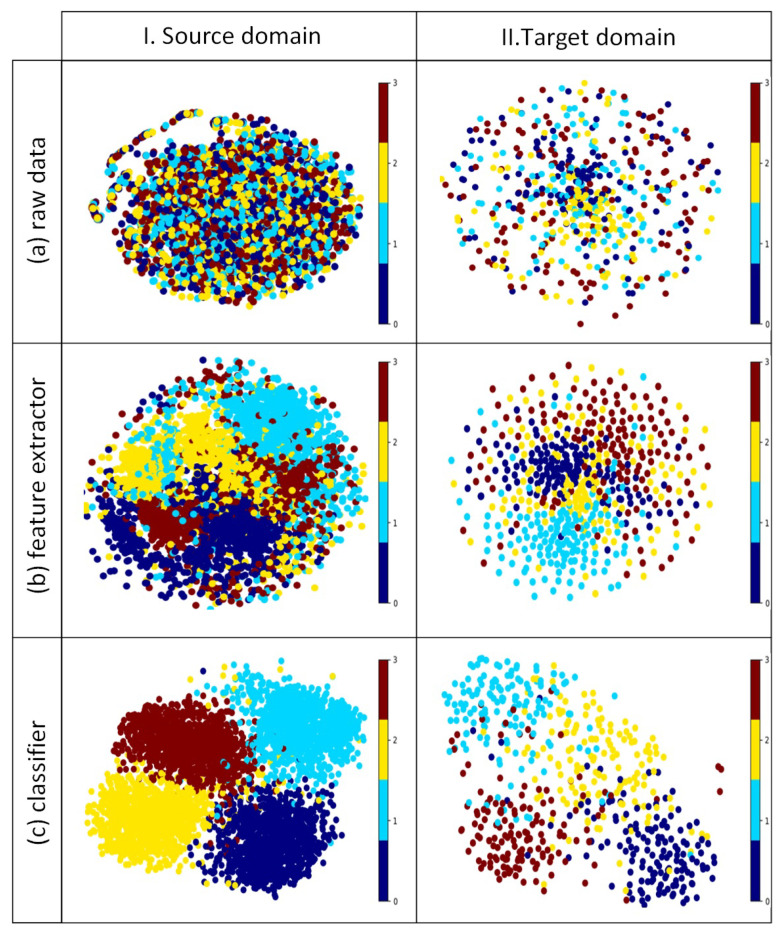
The feature maps obtained by t-SNE. Different colors denote different MI classification tasks. Part (**a**–**c**) is the data distribution of the different parts in the student network of the proposed model. Source domain I includes 8 subdomains, namely subjects 1–7 and 9, while the target domain comes from the 7th subject from the BCIC-IV-2a dataset.

**Figure 9:**
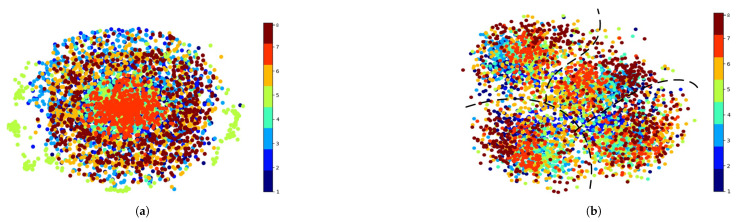
The feature maps obtained by t-SNE. Different colors denote 8 different subdomains, namely subjects 1–7 and 9, which are included in the source domain. (**a**) The data distribution of raw EEG signals. (**b**) Feature maps were extracted before the fully connected layer in the proposed model.

To better show the classification performance of the proposed model, we utilized the t-distributed stochastic neighbor embedding (t-SNE) tool to visualize the feature distribution of different parts in the student network of the proposed model. We used the data from subject 8 in the BCIC-IV-2a datadet as the target subject, while the other eight subjects were the source subjects. Figure 8 demonstrates the excellent generalization capability in decoding cross-subject MI-EEG signals without requiring access to unseen target data during the training process. Because the proposed model utilized the feature alignment method to acquire cross-domain knowledge, we also assessed the model’s feature aggregation performance in Figure 9. The t-SNE visualization in Figure 9a shows that different subdomains have different data distributions. Figure 9b was obtained before the fully connected layer in the classifier part of the student network, clearly demonstrating that the proposed model captures the invariant features of cross-domains and reduces the differences between cross-subjects. Black dashed lines divided the feature maps into four parts corresponding to four MI tasks in the BCIC-IV-2a dataset. In each part, features from different subdomains of the same label are effectively aggregated together, which further shows the superior classification and generalization ability of the proposed model.

### 3.8. Limitations

While our methodology demonstrated robust performance in offline dataset validation, the true challenge for practical BCI implementation lies in real-world deployment. Critical barriers emerge when transitioning from controlled environments to operational settings, such as computational bottlenecks in real-time EEG processing, inter-subject neural variability necessitating on-the-fly model adaptation, hardware-software compatibility constraints for portable systems, vulnerability to environmental perturbations (e.g., motion artifacts), and performance degradation caused by non-stationary neural dynamics. These challenges underscore the imperative need to develop latency-optimized inference architectures that harmonize accuracy with efficiency. Moving forward, we plan to systematically investigate adaptive calibration frameworks, artifact-resilient preprocessing schemes, and hardware-aware co-design paradigms to bridge this translational gap.

## 4. Discussion

In this work, we proposed a cross-subject model with domain generalization for MI-EEG classification. To obtain excellent decoding performance for each subject, the within-subject model was built with adequate samples from the same subject. However, high time-consuming calibration and data collection in the within-subject model training procedure limits the implementation of plug-and-play functionality for MI-BCI applications. Therefore, it is necessary to construct a cross-subject model using previously collected data, namely source domain data, to classify the target subject MI tasks without the need to collect target data. However, the variability in data distributions among different subjects within the source domain can lead to a decrease in the classification accuracy of cross-subject models. Previous studies [29] adopted the approach of collecting a small portion of data exclusively from the target subjects and using adaptive methods based on models trained on the source domain to improve the performance of cross-subject models. However, this DA-based approach still necessitates conducting additional experiments to acquire electroencephalogram (EEG) data from target subjects, essentially leading to the creation of a new model for each new subject. DG-based approaches train a generalized model through training on multiple datasets in the source domain, enabling it to exhibit strong performance on an unseen domain.

In the proposed model, we employed a domain-invariant feature learning strategy to learn representations that maintain invariance across domains. The invariant features consist of two sides, namely, internal and mutual sides. The internally invariant features allow the model to focus on the spectral features corresponding to MI tasks. We utilized a knowledge distillation framework and trained the teacher and student networks, respectively. The teacher network comprises the spectral features fusion block, feature extractor, and classifier, whereas the student network consists solely of the feature extractor and the classifier. We used the pointwise convolution to adopt cross-frequency interactions corresponding to the MI information, which proves useful for enhancing the robustness of spectral representation. Then, in the feature extractor, temporal–spatial convolution was employed to capture the discriminative features in MI EEG. The inclusion of two dense units, creating short connections within the CNN layers, facilitated feature refinement and the extraction of more abstract characteristics. To transfer the internally invariant spectral features from the teacher network to the student network, we employed MSE loss to encourage the student network’s features to closely align with those of the teacher network. For mutually invariant features that are exploited from different subdomains, we used the correlation alignment method to align the data distribution and learn the cross-domain transferable knowledge. To eliminate the redundancy and repeated information among two kinds of features, we used distance regularization to maximize their differences. To better validate the superiority of the proposed model, we conducted experiments on two public datasets. Based on the data presented in Table 2 and Table 3, it is evident that our proposed model outperformed the state-of-the-art methods, attaining the highest classification performance. The ablation study in Table 4 also demonstrates the usage of two kinds of invariant features and the effect of distance regularization. The visualization results based on t-SNE in Figure 8 present the MI-EEG decoding performance of the proposed model. The feature maps obtained in the classifier based on source subjects exhibit very distinct clusters, effectively showing the feature distribution of different labels. Even though the target subject is the unseen domain, the proposed model can effectively classify MI tasks by utilizing acquired generalized information and applying it in a plug-and-play BCI system.

Although our suggested model has demonstrated better performance compared to prior approaches, there remains potential for enhancement. Firstly, the hyper-parameters used in the loss function are adjusted manually. The parameter sensitivity in different datasets is different, according to Figure 5 and Figure 6. Future work should enable the autonomous learning and optimization of hyperparameters within the model. Secondly, real-world problems will not only encounter variations across subjects but also practical demands across different scenarios and devices. Models should not only learn domain-invariant features at high-level abstraction but also perform optimization and weight redistribution learning across channels or time periods. Thirdly, we only test the model performance and visualize the feature distribution. In the future, we can employ interpretable techniques in deep learning models to explain invariant features and propose their specific physical meanings, mutually corroborating them with relevant neural mechanisms.

## 5. Conclusions

Our study demonstrates significant advancements in cross-subject motor imagery EEG decoding through a novel domain generalization framework that enables plug-and-play BCI functionality by learning both internally invariant spectral-task relationships via knowledge distillation and mutually invariant cross-domain representations through correlation alignment, further enhanced by distance regularization to maximize generalized feature expression, achieving state-of-the-art classification accuracy improvements of 8.93% on BCIC-IV-2a and 4.4% on KU datasets compared to existing deep learning methods, with feature distribution analyses confirming superior generalization capabilities across unseen subjects.

## Figures and Tables

**Table 1:** The detailed architecture of the teacher network.

Block	Layer	Filters	Size	Output	Activation	Options
Spectral feature fusion	Input			(1, C, T)		
	Concatenate (filtered)			(N, C, T)		
	Pointwise Conv 2D	1	(1, 1)	(1, C, T)	Linear	
Feature extractor	Conv 2D	F1	(1, C1)	(F1, C, T)	Linear	padding = same
	Batch Normalization					
	Depthwise Conv 2D	D × F1	(C, 1)	(F1, 1, T)	ELU	padding = same, depth = D
	Batch Normalization					
(Dense Unit 1)	Conv 2D	F2	(1, C2)	(F1 + F2, 1, T)	ELU	padding = same
	Batch Normalization					
	Dropout					
	Conv 2D	F2	(1, C2)	(F1 + 2 × F2, 1, T)	ELU	padding = same
	Batch Normalization					
	Dropout					
	Conv 2D	F2	(1, C2)	(F1 + 3 × F2, 1, T)	ELU	padding = same
	Batch Normalization					
	Dropout					
	Average Pooling		(1, 5)	(F1 + 3 × F2, 1, T // 5)		
(Dense Unit 2)		F2	(1, C3)	(F1 + 6 × F2, 1, T // 25)		
Classifier	Conv 1D	F3	(1, 1)	(F3, 1, T // 25)	ELU	
	Flatten					
	Dense	N × (F3 × T // 25)		N	Softmax	max norm = 0.25

**Table 2:** Comparison of average classification accuracy (%) and standard deviation (Std) on the BCIC-IV-2a dataset.

Subject	CSP	FBCSP	Shallow ConvNet	EEGNet	FBCNet	Proposed Model
1	32.36	42.5	70.78	54.83	49.55	74.65
2	25.8	26.27	37.73	30.94	31.02	44.96
3	35.82	51.49	64.65	60.38	58.68	64.06
4	33.23	31.88	47.97	38.87	41.41	51.73
5	24.91	26.51	29.25	28.8	28.3	52.95
6	26.15	27.01	33.82	26.64	32.17	44.44
7	28.96	23.65	44.58	32.03	28.58	69.27
8	49.53	51.37	70.78	63.29	51.25	74.3
9	32.03	38.35	60.68	54.96	50.49	64.23
Avg	32.09 **	35.45 **	51.14 *	43.42 **	41.27 **	60.07
Std	7.55	10.93	16.04	14.78	11.58	11.86

* and ** denote the statistical significance between the classification results of the proposed model and the baseline models with *: *p* < 0.05 and **: *p* < 0.01.

**Table 3:** Comparison of average classification accuracy (%) and standard deviation (Std) on the KU dataset.

	CSP	FBCSP	Shallow ConvNet	EEGNet	FBCNet	Dynamic Shallow ConvNet	Dynamic EEGNet	Dynamic EEGInception	Proposel Model
Avg	56.08 **	65.19 **	74.62 **	72.23 **	71.54 **	70.30 **	71.90 **	77.40 **	81.80
Std	6.82	13.04	12.15	13.93	14.07	11.10	12.10	10.00	10.70

** denote the statistical significance between the classification results of the proposed model and the baseline models with **: *p* < 0.01.

**Table 4:** Ablation study of the proposed model. Comparison of average classification accuracy (%) and standard deviation (SD) of the BCIC-IV-2a and KU datasets.

	BCIC-IV-2a (SD)	KU (SD)
w./o Inter	54.61 (10.31)	81.00 (11.12)
w./o Mutual	57.50 (12.28)	80.52 (11.09)
w./o Div	56.19 (12.61)	75.85 (9.34)
w./o General	55.12 (12.00)	79.32 (10.56)
Proposed model	60.07 (11.86)	81.80 (10.70)

## Data Availability

The original contributions presented in the study are included in the article, further inquiries can be directed to the corresponding authors.
